# Supplementary material for: Specificity, length, and luck: How genes are prioritized by rare and common variant association studies
Source: bioRxiv. 2024 Dec 16:2024.12.12.628073. Preprint. [Version 1] doi: 10.1101/2024.12.12.628073 (PMC11812597; doi:10.1101/2024.12.12.628073)
Supplement: Supplement 2 [file NIHPP2024.12.12.628073v1-supplement-2.pdf]

bioRxiv preprint doi: https://doi.org/10.1101/2024.12.12.628073; this version posted December 16, 2024. The copyright holder for this preprint (which was not certified by peer review) is the author/funder, who has granted bioRxiv a license to display the preprint in perpetuity. It is made available under aCC-BY 4.0 International license.

Supplementary Table

Supplementary Table 1: **List of traits and abbreviations used in the study.** *Table of the 209 traits used in this study with the UKB trait IDs, trait names, abbreviations used, tissue to which each trait was linked (if applicable), and an indication of whether or not the trait was included in our subset of 27 genetically uncorrelated traits (Methods).*

bioRxiv preprint doi: https://doi.org/10.1101/2024.12.12.628073; this version posted December 16, 2024. The copyright holder for this preprint (which was not certified by peer review) is the author/funder, who has granted bioRxiv a license to display the preprint in perpetuity. It is made available under aCC-BY 4.0 International license.

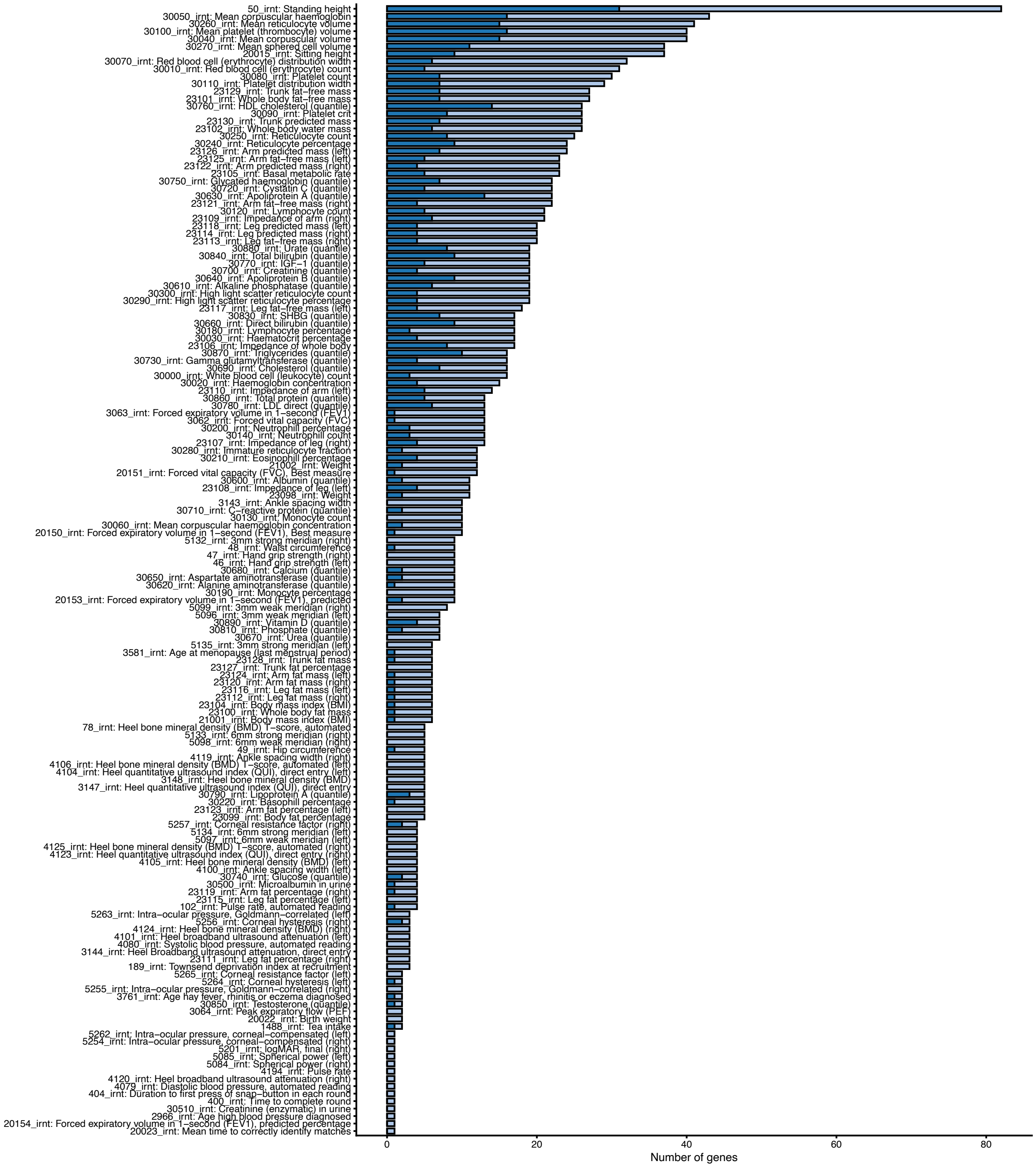

Figure S1: **Limited GWAS overlap at top LoF burden hits.**

*Extended version of Figure 1**C**, including all traits with at least one genome-wide significant LoF burden test. Dark blue bars correspond to genome-wide significant LoF burden test genes that also overlap a top GWAS locus (Methods). Light blue bars are genome-wide significant LoF burden test genes that do not overlap a top GWAS locus.*

bioRxiv preprint doi: https://doi.org/10.1101/2024.12.12.628073; this version posted December 16, 2024. The copyright holder for this preprint (which was not certified by peer review) is the author/funder, who has granted bioRxiv a license to display the preprint in perpetuity. It is made available under aCC-BY 4.0 International license.

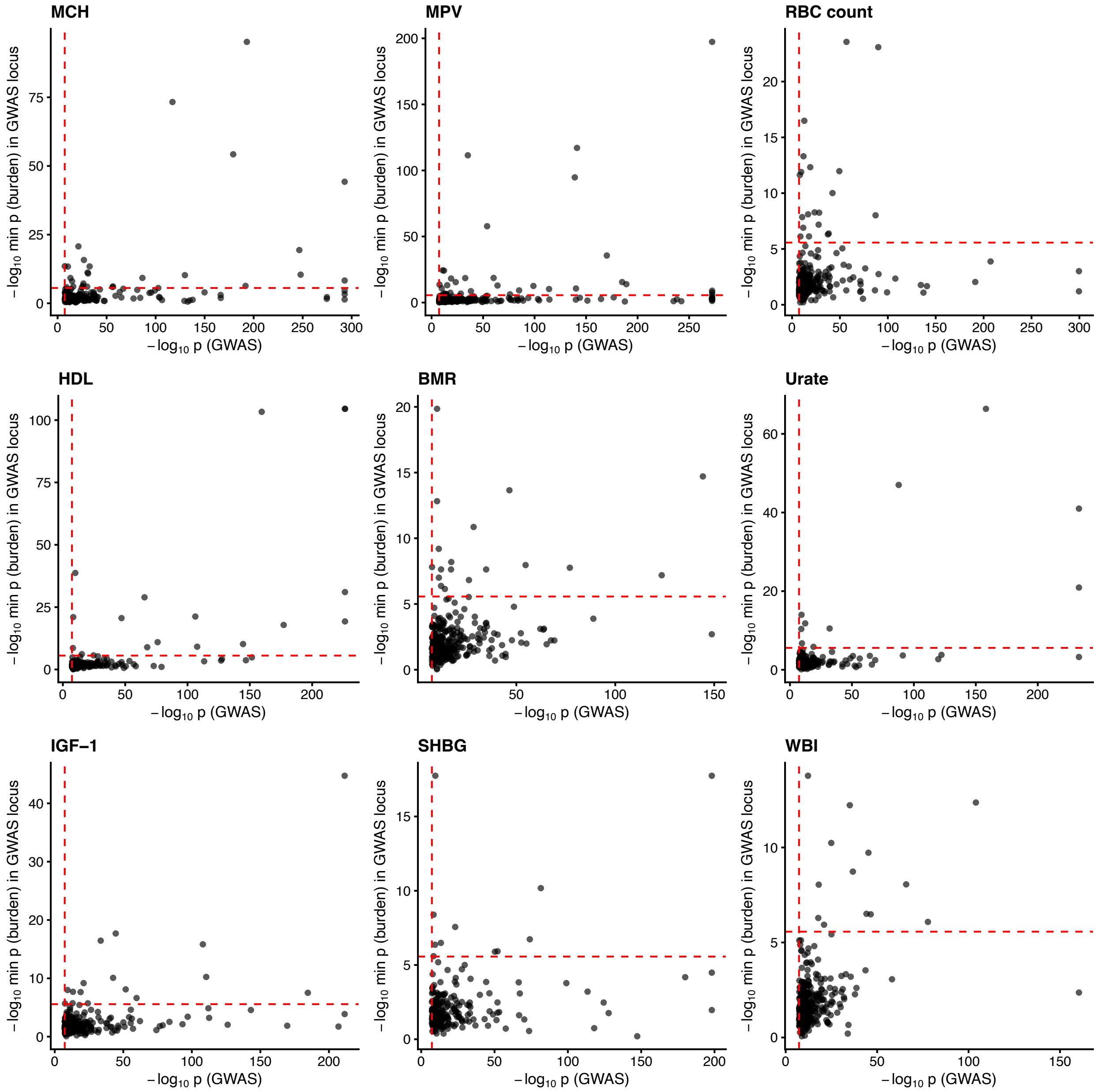

Figure S2: **Strongest GWAS hits are not always strong LoF burden hits.**
*Extended version of Figure 1**D**, including 9 additional traits. Each point is a significant GWAS locus (Methods). Dashed red lines are thresholds for genome-wide significance.*

bioRxiv preprint doi: https://doi.org/10.1101/2024.12.12.628073; this version posted December 16, 2024. The copyright holder for this preprint (which was not certified by peer review) is the author/funder, who has granted bioRxiv a license to display the preprint in perpetuity. It is made available under aCC-BY 4.0 International license.

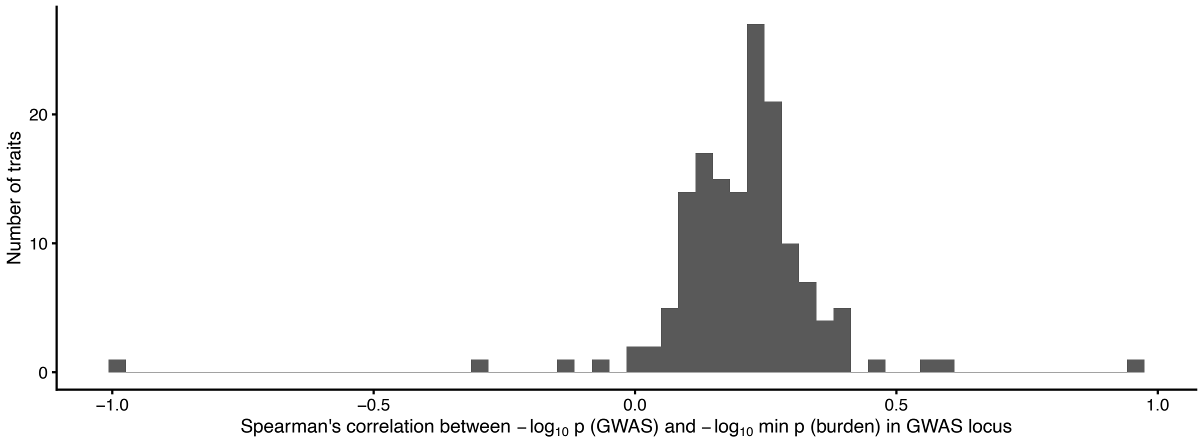

Figure S3: **Modest correlation between GWAS and LoF burden test p-value ranks.**
Histogram of Spearman's ρ *between the minimum GWAS* $-\log_{10}$ *p-value of any variant within a given GWAS locus and the minimum LoF burden* $-\log_{10}$ *p-value of any gene overlapping that locus. By definition all GWAS loci contain at least one genome-wide significant variant (Methods).*

bioRxiv preprint doi: https://doi.org/10.1101/2024.12.12.628073; this version posted December 16, 2024. The copyright holder for this preprint (which was not certified by peer review) is the author/funder, who has granted bioRxiv a license to display the preprint in perpetuity. It is made available under aCC-BY 4.0 International license.

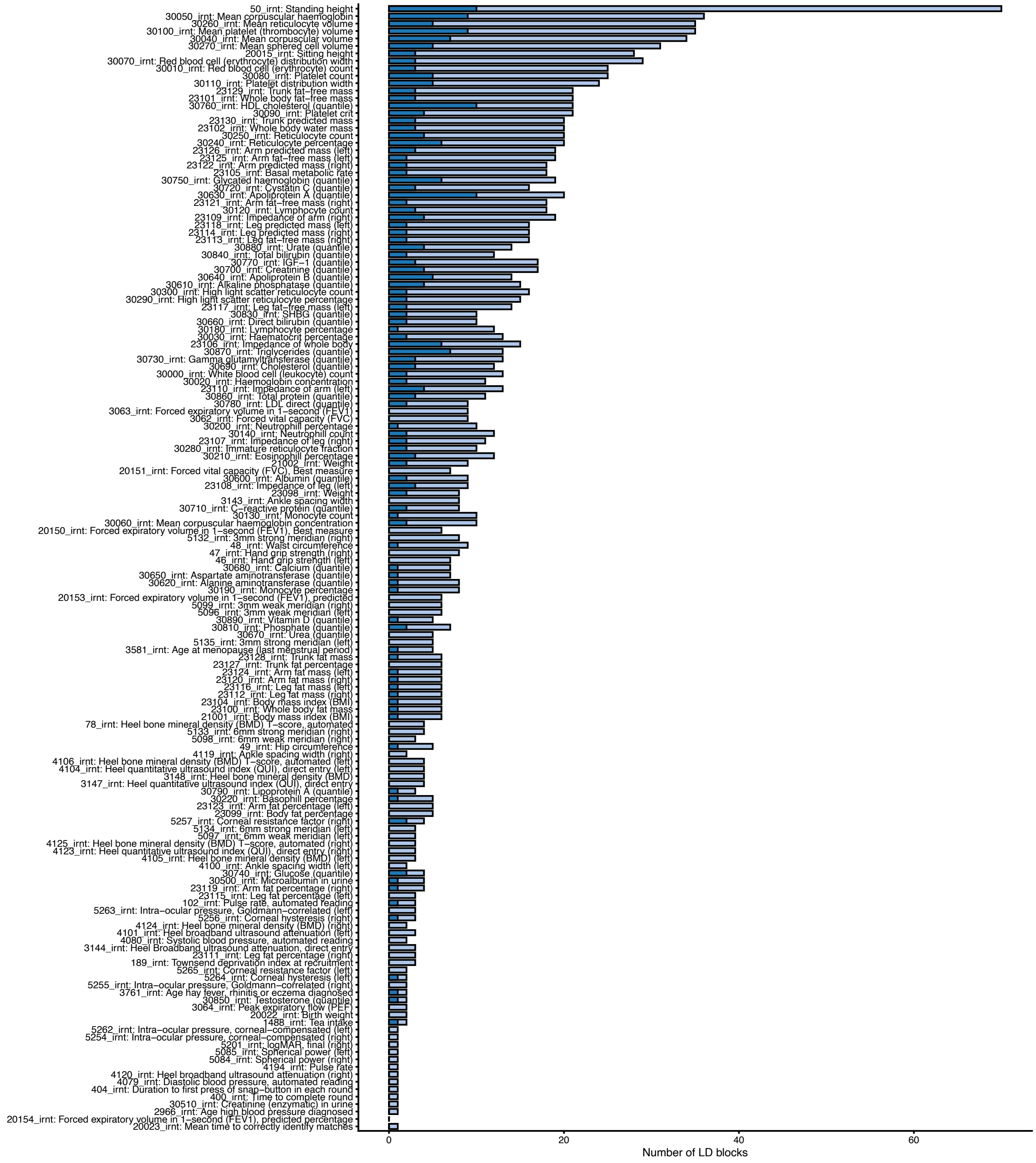

Figure S4: **Limited GWAS overlap at top LoF burden LD blocks.**
Alternate version of Supplementary Figure S1 but using LD blocks instead of GWAS loci. Dark blue bars correspond to LD blocks that contain a genome-wide significant LoF burden test gene that are also top LD blocks for GWAS. Light blue bars are LD blocks containing genome-wide significant LoF burden test genes that are not also top GWAS LD blocks.

bioRxiv preprint doi: https://doi.org/10.1101/2024.12.12.628073; this version posted December 16, 2024. The copyright holder for this preprint (which was not certified by peer review) is the author/funder, who has granted bioRxiv a license to display the preprint in perpetuity. It is made available under aCC-BY 4.0 International license.

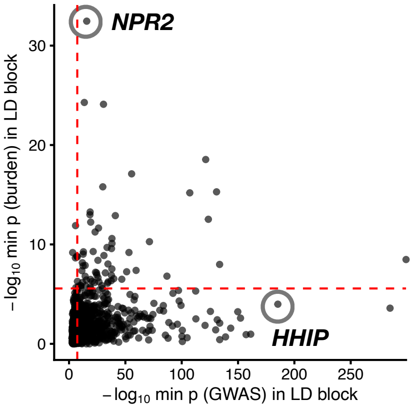

Figure S5: **GWAS and LoF burden tests prioritize different LD blocks for height.**
*Alternate version of Figure 1***D** *but using LD blocks instead of GWAS loci. Each point is an LD block. Dashed red lines are thresholds for genome-wide significance.*

bioRxiv preprint doi: https://doi.org/10.1101/2024.12.12.628073; this version posted December 16, 2024. The copyright holder for this preprint (which was not certified by peer review) is the author/funder, who has granted bioRxiv a license to display the preprint in perpetuity. It is made available under aCC-BY 4.0 International license.

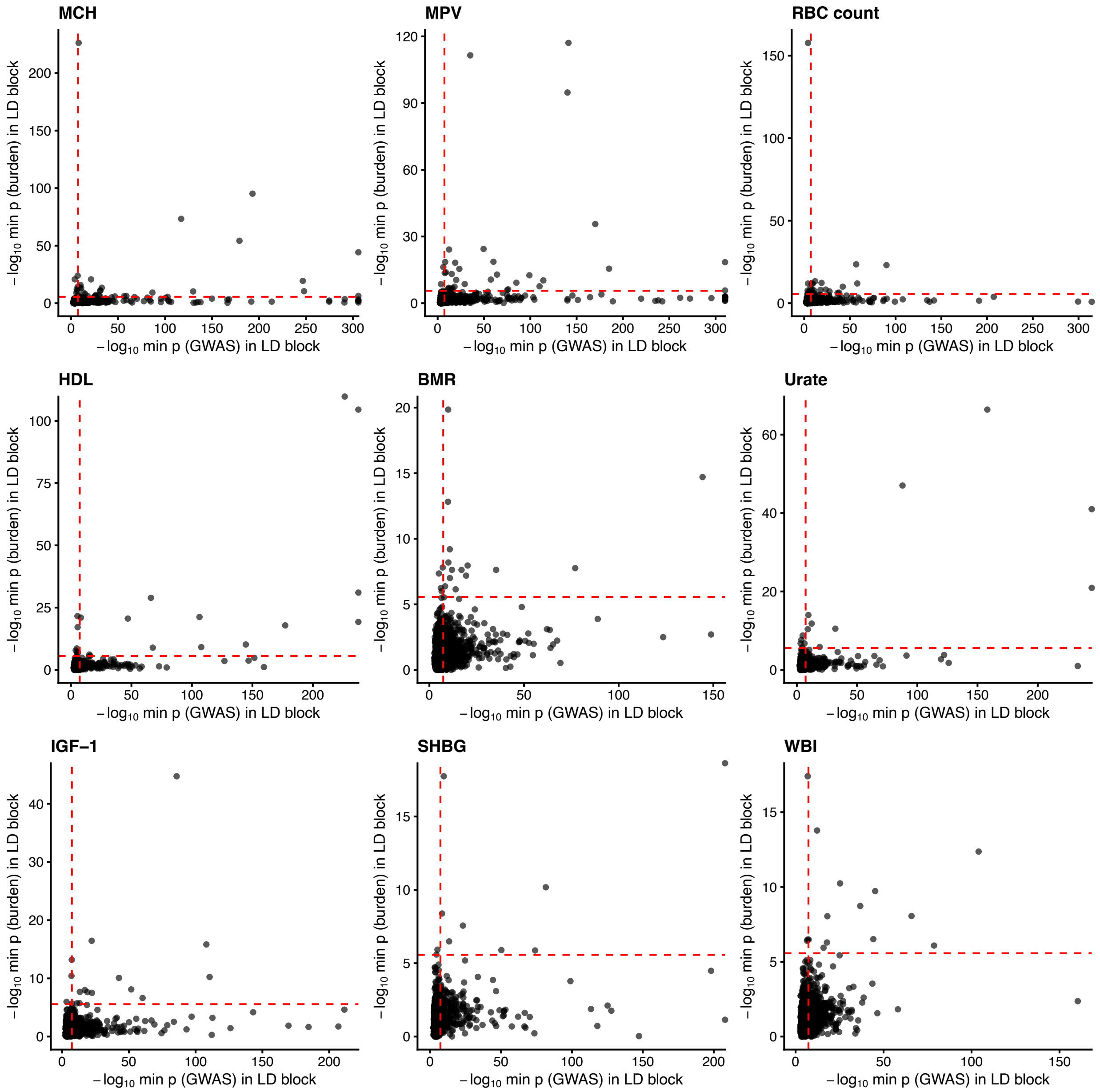

Figure S6: **GWAS and LoF burden tests prioritize different LD blocks across traits.**
Alternate version of Supplementary Figure S2 but using LD blocks instead of GWAS loci. Each point is an LD block. Dashed red lines are thresholds for genome-wide significance.

bioRxiv preprint doi: https://doi.org/10.1101/2024.12.12.628073; this version posted December 16, 2024. The copyright holder for this preprint (which was not certified by peer review) is the author/funder, who has granted bioRxiv a license to display the preprint in perpetuity. It is made available under aCC-BY 4.0 International license.

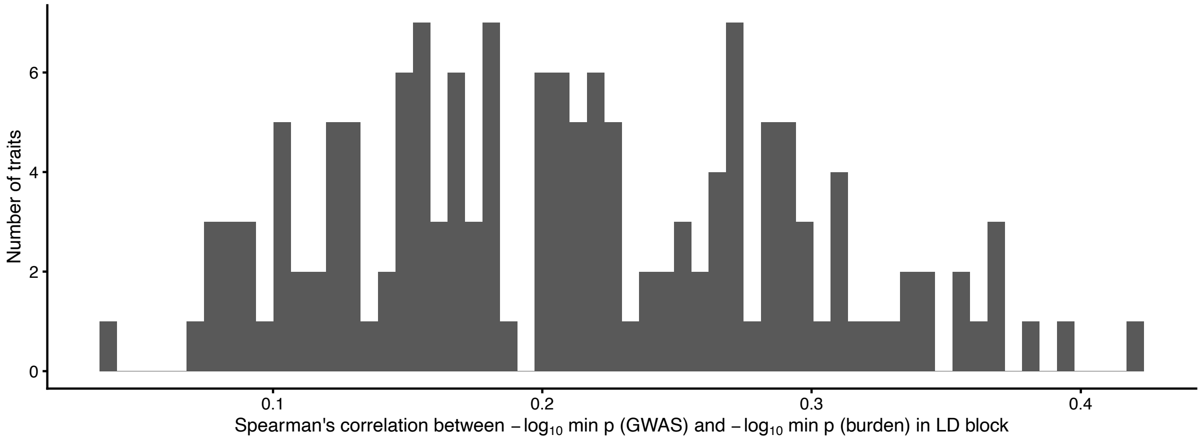

Figure S7: **Modest correlation between GWAS and LoF burden test p-value ranks across LD blocks.**

Alternate version of Supplementary Figure S3 but using LD blocks instead of GWAS loci. Histogram of Spearman's ρ *between the minimum GWAS* $-\log_{10}$ *p-value and the minimum LoF burden* $-\log_{10}$ *p-value across LD blocks.*

bioRxiv preprint doi: https://doi.org/10.1101/2024.12.12.628073; this version posted December 16, 2024. The copyright holder for this preprint (which was not certified by peer review) is the author/funder, who has granted bioRxiv a license to display the preprint in perpetuity. It is made available under aCC-BY 4.0 International license.

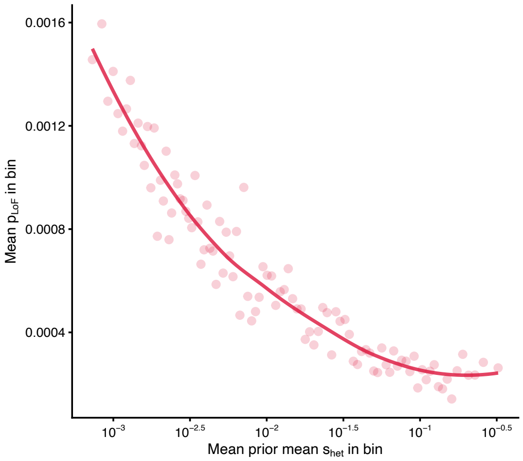

Figure S8: p_{LoF} **and** $s_{\mathbf{het}}$ **are negatively correlated.**
*Alternate version of Figure 3**B** but binning genes by the prior mean s_{het} as reported by [33]. These estimates are learned using GeneBayes [33], which uses frequency data across genes to learn a function mapping gene features (e.g., expression patterns across tissues) to a prior on s_{het}. In the main text, we used GeneBayes posterior mean estimates, which use this learned prior for each gene along with that gene's p_{LoF} to estimate s_{het}. Here we use the prior mean, which uses the p_{LoF} data across genes to learn per-gene priors, but does not use a gene's p_{LoF} when estimating its s_{het}.*

bioRxiv preprint doi: https://doi.org/10.1101/2024.12.12.628073; this version posted December 16, 2024. The copyright holder for this preprint (which was not certified by peer review) is the author/funder, who has granted bioRxiv a license to display the preprint in perpetuity. It is made available under aCC-BY 4.0 International license.

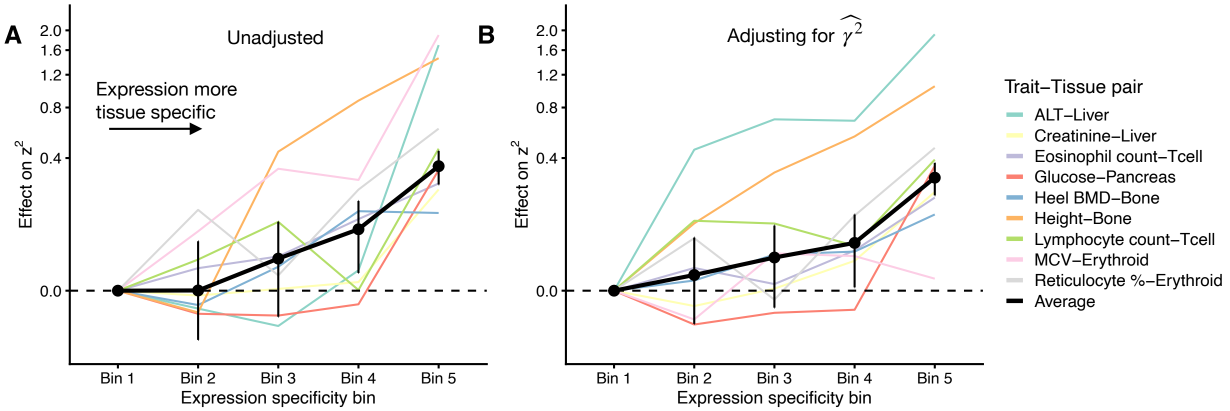

Figure S9: **Expression specificity increases LoF burden test z-scores.**
For 9 trait-tissue pairs we regressed LoF burden test z^2 for each gene on either **A**) *expression specificity bin or* **B**) *expression specificty bin and an unbiased estimate of γ^2, $\widehat{\gamma^2}$. Since the 5 bins are co-linear, we report all regression coefficients relative to the effect in expression specificity bin 1. Colored lines are regression coefficients for individual trait-tissue pairs. The black line is the inverse variance-weighted average across trait-tissue pairs. The y-axes have been non-linearly transformed.*

bioRxiv preprint doi: https://doi.org/10.1101/2024.12.12.628073; this version posted December 16, 2024. The copyright holder for this preprint (which was not certified by peer review) is the author/funder, who has granted bioRxiv a license to display the preprint in perpetuity. It is made available under aCC-BY 4.0 International license.

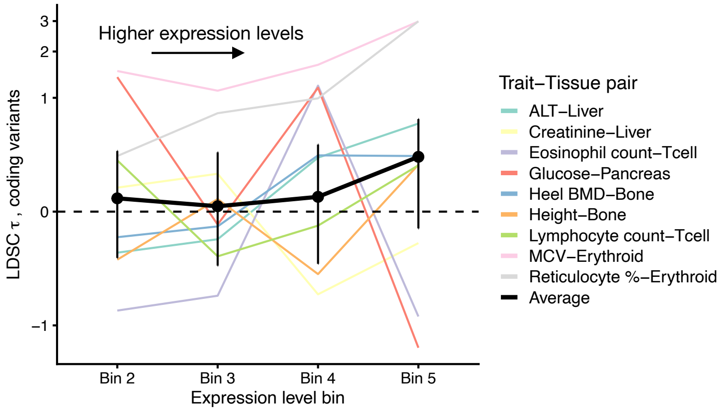

Figure S10: **Expression levels do not play a large role in GWAS coding heritability.**
S-LDSC analysis results for 9 trait-tissue pairs. Result are reported in terms of τ, a measure of heritability enrichment. Variants are binned by the expression level (as measured by TPM) of the corresponding gene. Since the 5 bins are co-linear, we drop the bin 1 annotation and only report results for the remaining bins. These results are from a joint analysis including both expression specificity and expression level bins as covariates. Colored lines are τ estimates for individual trait-tissue pairs. The black line is the inverse variance-weighted average across trait-tissue pairs. The y-axis has been non-linearly transformed.

bioRxiv preprint doi: https://doi.org/10.1101/2024.12.12.628073; this version posted December 16, 2024. The copyright holder for this preprint (which was not certified by peer review) is the author/funder, who has granted bioRxiv a license to display the preprint in perpetuity. It is made available under aCC-BY 4.0 International license.

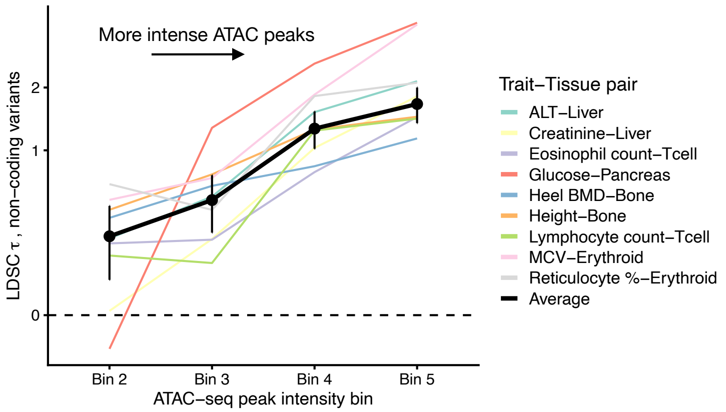

Figure S11: **ATAC peak intensity increases heritability explained.**
S-LDSC analysis results for 9 trait-tissue pairs. Result are reported in terms of τ, a measure of heritability enrichment. Variants are binned by the intensity of their ATAC-seq peaks (Methods). Since the 5 bins are co-linear, we drop the bin 1 annotation and only report results for the remaining bins. These results are from a joint analysis including both ATAC specificity and ATAC intensity bins as covariates. Colored lines are the τ estimates for individual trait-tissue pairs. The black line is the inverse variance-weighted average across trait-tissue pairs. Note that the y-axis has been non-linearly transformed.

bioRxiv preprint doi: https://doi.org/10.1101/2024.12.12.628073; this version posted December 16, 2024. The copyright holder for this preprint (which was not certified by peer review) is the author/funder, who has granted bioRxiv a license to display the preprint in perpetuity. It is made available under aCC-BY 4.0 International license.

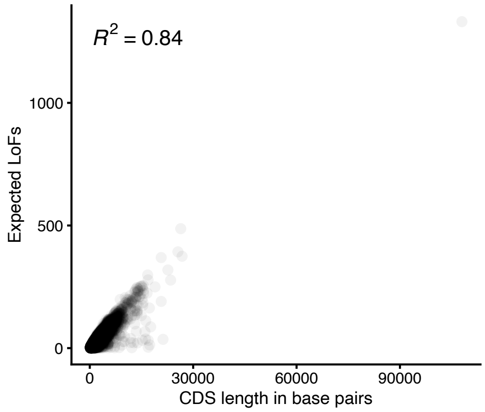

Figure S12: **CDS length and expected number of unique LoFs are highly correlated.**
Scatter plot of the expected length in base pairs for the canonical CDS for each gene (Methods) and the expected number of unique LoFs as computed by gnomAD [43]. The overall correlation is high (Pearson's $r^2 = 0.84$*).*

bioRxiv preprint doi: https://doi.org/10.1101/2024.12.12.628073; this version posted December 16, 2024. The copyright holder for this preprint (which was not certified by peer review) is the author/funder, who has granted bioRxiv a license to display the preprint in perpetuity. It is made available under aCC-BY 4.0 International license.

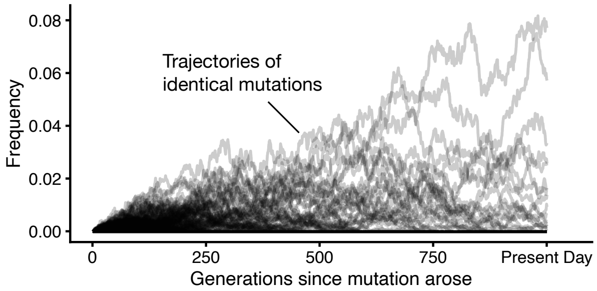

Figure S13: **Genetic drift generates variance in allele frequencies.**
10,000 frequency trajectories of identical mutations simulated under the Discrete-Time Wright-Fisher model. Trajectories were simulated assuming no mutation, an s_{het} of 10^{-3}, no fitness consequences in homozygotes, and a population size of $N_e = 10{,}000$. All mutations were assumed to arise 1,000 generations before present.

bioRxiv preprint doi: https://doi.org/10.1101/2024.12.12.628073; this version posted December 16, 2024. The copyright holder for this preprint (which was not certified by peer review) is the author/funder, who has granted bioRxiv a license to display the preprint in perpetuity. It is made available under aCC-BY 4.0 International license.

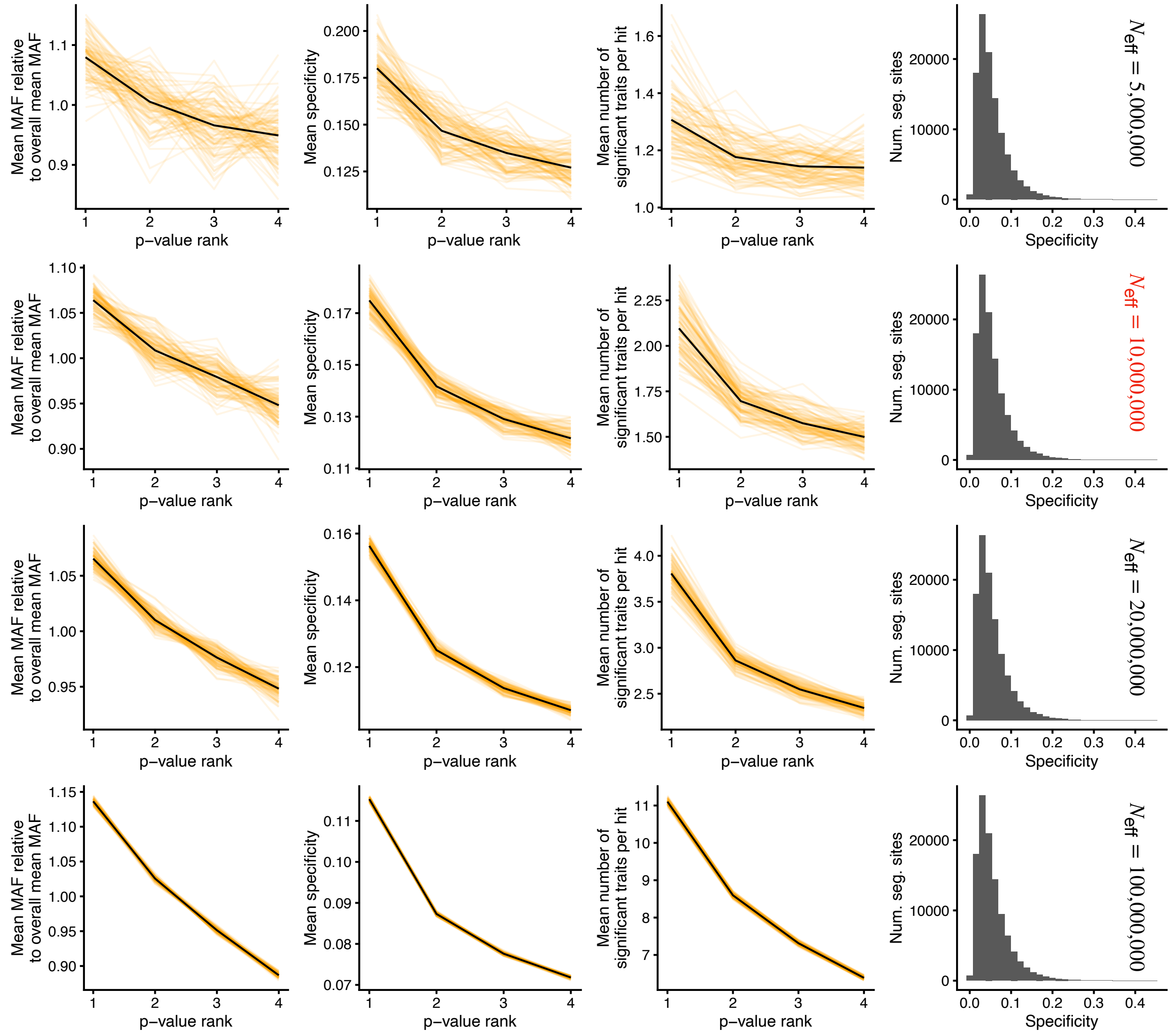

Figure S14: **Robustness of apparent pleiotropy to simulation parameter $N_{\mathbf{eff}}$.**
*Analogous to Figure 5**F-H**, but with varying N_{eff} (see Methods for definition), while holding all other simulation parameters fixed to the values used in the main text. Results from individual population genetic simulations are in orange, and the mean across simulations is in black. The histograms show the distribution of trait specificity, Ψ_V, across segregating sites for a single simulation. N_{eff} does not affect the distribution of effect sizes, and so these are the same across values of N_{eff}. The value of N_{eff} used in the main text is in red.*

bioRxiv preprint doi: https://doi.org/10.1101/2024.12.12.628073; this version posted December 16, 2024. The copyright holder for this preprint (which was not certified by peer review) is the author/funder, who has granted bioRxiv a license to display the preprint in perpetuity. It is made available under aCC-BY 4.0 International license.

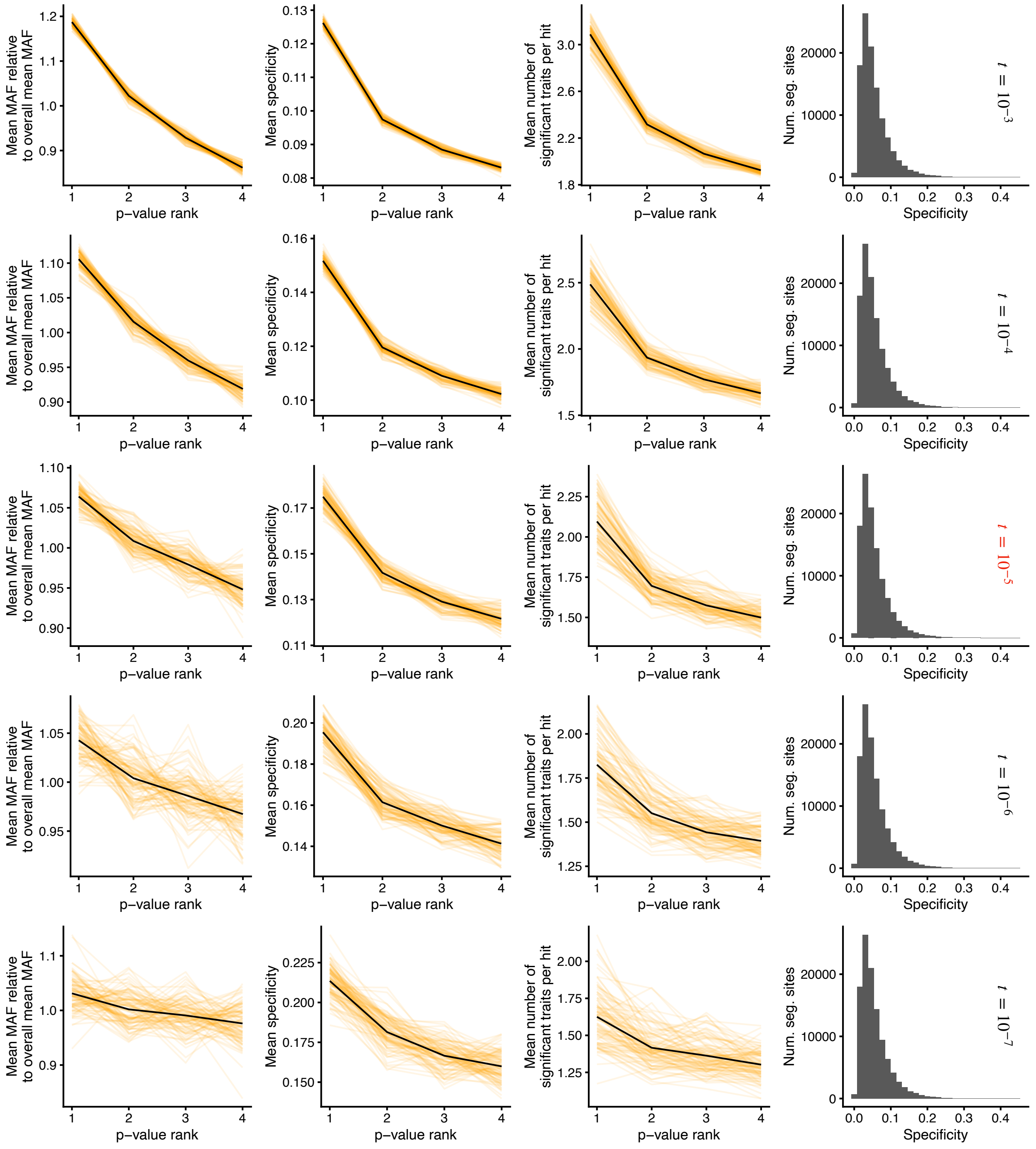

Figure S15: **Robustness of apparent pleiotropy to simulation parameter t.**

*Analogous to Figure 5**F**-**H**, but with varying t (see Methods for definition), while holding all other simulation parameters fixed to the values used in the main text. Results from individual population genetic simulations are in orange, and the mean across simulations is in black. The histograms show the distribution of trait specificity, Ψ_V, across segregating sites for a single simulation. t does not affect the distribution of effect sizes, and so these are the same across values of t. The value of t used in the main text is in red.*

bioRxiv preprint doi: https://doi.org/10.1101/2024.12.12.628073; this version posted December 16, 2024. The copyright holder for this preprint (which was not certified by peer review) is the author/funder, who has granted bioRxiv a license to display the preprint in perpetuity. It is made available under aCC-BY 4.0 International license.

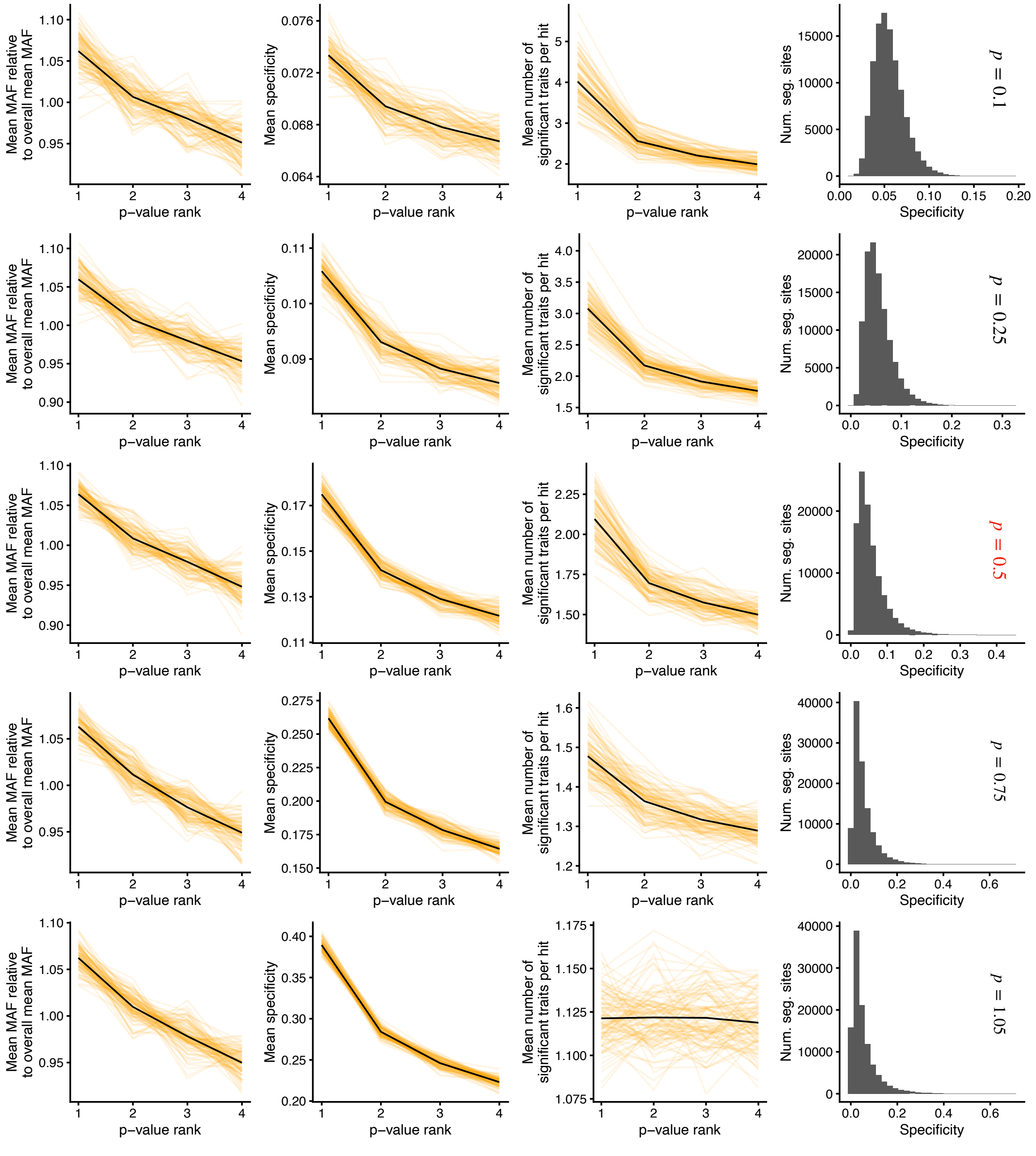

Figure S16: **Robustness of apparent pleiotropy to simulation parameter p.**
*Analogous to Figure 5**F-H**, but with varying p (see Methods for definition), while holding all other simulation parameters fixed to the values used in the main text. Results from individual population genetic simulations are in orange, and the mean across simulations is in black. The histograms show the distribution of trait specificity, Ψ_V, across segregating sites for a single simulation. The value of p used in the main text is in red.*

bioRxiv preprint doi: https://doi.org/10.1101/2024.12.12.628073; this version posted December 16, 2024. The copyright holder for this preprint (which was not certified by peer review) is the author/funder, who has granted bioRxiv a license to display the preprint in perpetuity. It is made available under aCC-BY 4.0 International license.

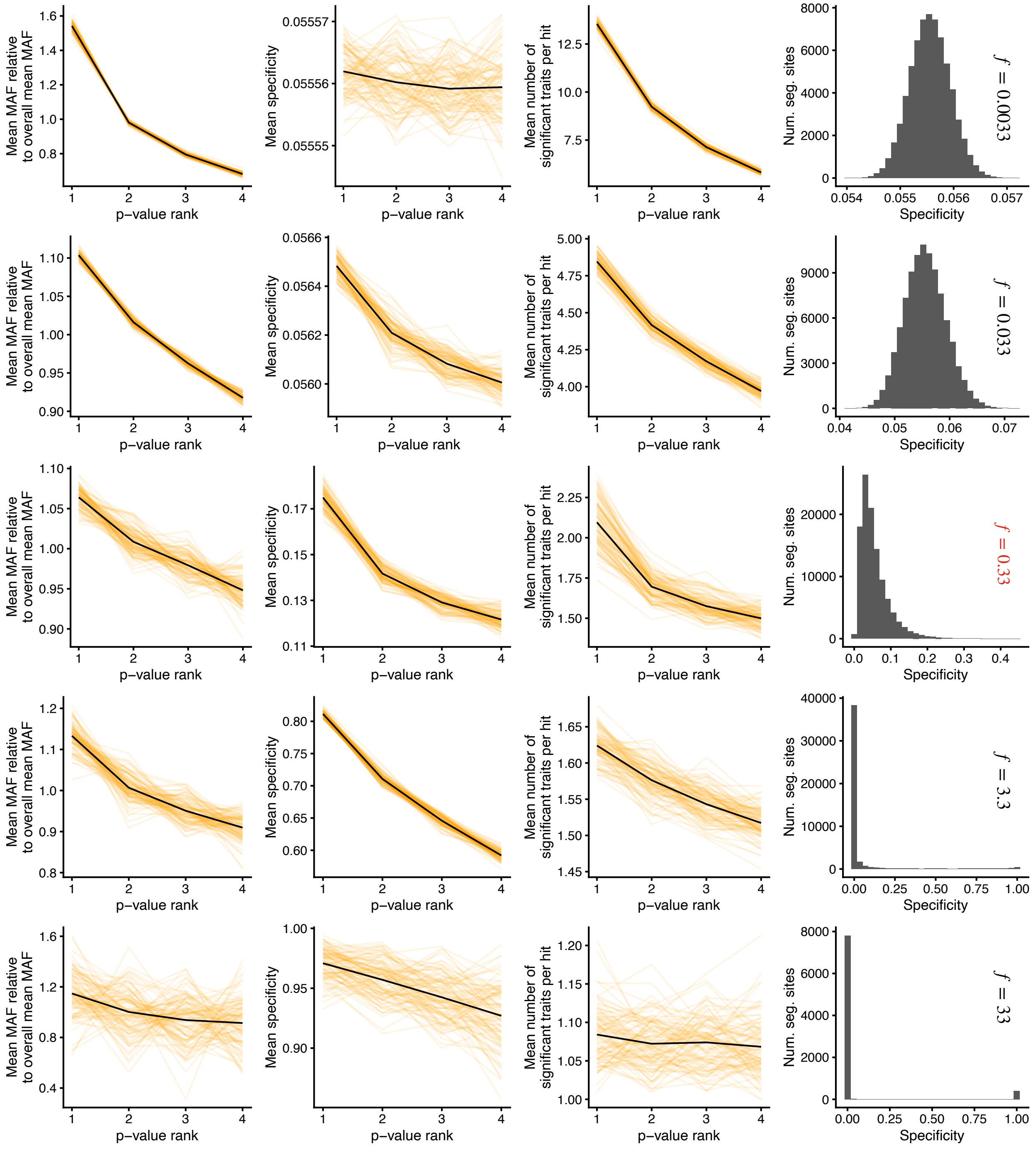

Figure S17: **Robustness of apparent pleiotropy to simulation parameter f.**

*Analogous to Figure 5**F-H**, but with varying f (see Methods for definition), while holding all other simulation parameters fixed to the values used in the main text. Results from individual population genetic simulations are in orange, and the mean across simulations is in black. The histograms show the distribution of trait specificity, Ψ_V, across segregating sites for a single simulation. The value of f used in the main text is in red.*

bioRxiv preprint doi: https://doi.org/10.1101/2024.12.12.628073; this version posted December 16, 2024. The copyright holder for this preprint (which was not certified by peer review) is the author/funder, who has granted bioRxiv a license to display the preprint in perpetuity. It is made available under aCC-BY 4.0 International license.

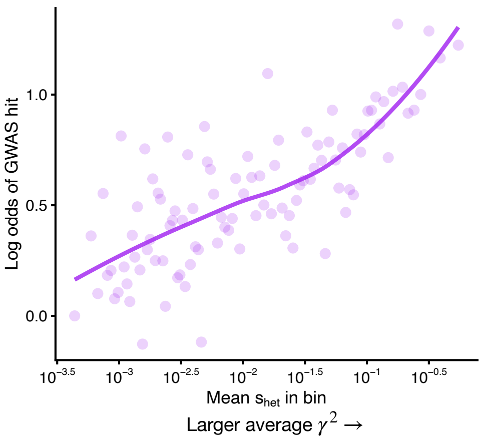

Figure S18: **Probability of a variant being a GWAS hit is correlated with s_{het}.**
Logistic regression coefficients for s_{het} percentile categories in a model that predicts whether a variant is a GWAS hit or not including various covariates such as distance to transcription start site (Methods). Each bin contains approximately 184 genes. The trend line is fit using LOESS.

bioRxiv preprint doi: https://doi.org/10.1101/2024.12.12.628073; this version posted December 16, 2024. The copyright holder for this preprint (which was not certified by peer review) is the author/funder, who has granted bioRxiv a license to display the preprint in perpetuity. It is made available under aCC-BY 4.0 International license.

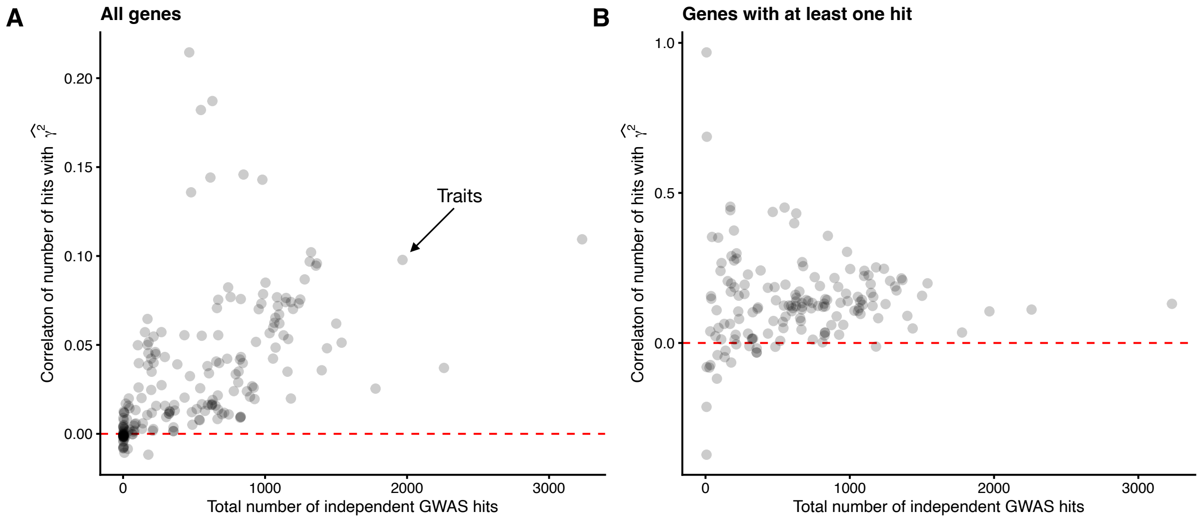

Figure S19: **Number of GWAS hits is predictive of γ^2.**
Scatter plots of the correlation across genes between the number of independent GWAS hits and an unbiased estimate of γ^2, $\widehat{\gamma^2}$, against the total number of independent GWAS hits. **A**) *Correlation across all genes. Traits with more independent hits tend to have a higher correlation between number of hits and $\widehat{\gamma^2}$.* **B**) *To make sure that the correlations in panel* **A** *were not driven just by presence or absence of any GWAS hits, we computed correlations between number of GWAS hits and $\widehat{\gamma^2}$ for only those genes with at least one GWAS hit. In both panels, it should be noted that $\widehat{\gamma^2}$ is generally a very noisy estimate of γ^2. This will drive the plotted correlations to be much lower than the true correlation between the number of GWAS hits and the unobserved true values of γ^2.*

bioRxiv preprint doi: https://doi.org/10.1101/2024.12.12.628073; this version posted December 16, 2024. The copyright holder for this preprint (which was not certified by peer review) is the author/funder, who has granted bioRxiv a license to display the preprint in perpetuity. It is made available under aCC-BY 4.0 International license.

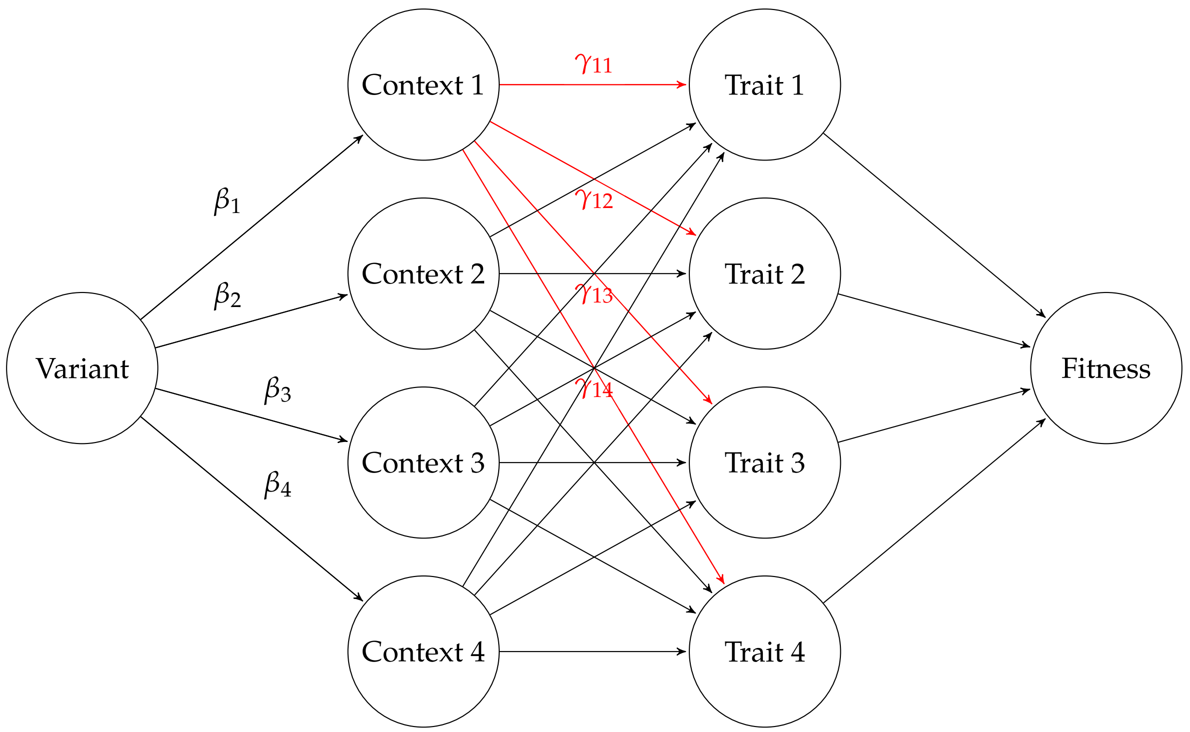

Figure S20: The most general model we will consider. A variant has effects in different contexts, and a gene determines how each context affects each trait.

bioRxiv preprint doi: https://doi.org/10.1101/2024.12.12.628073; this version posted December 16, 2024. The copyright holder for this preprint (which was not certified by peer review) is the author/funder, who has granted bioRxiv a license to display the preprint in perpetuity. It is made available under aCC-BY 4.0 International license.

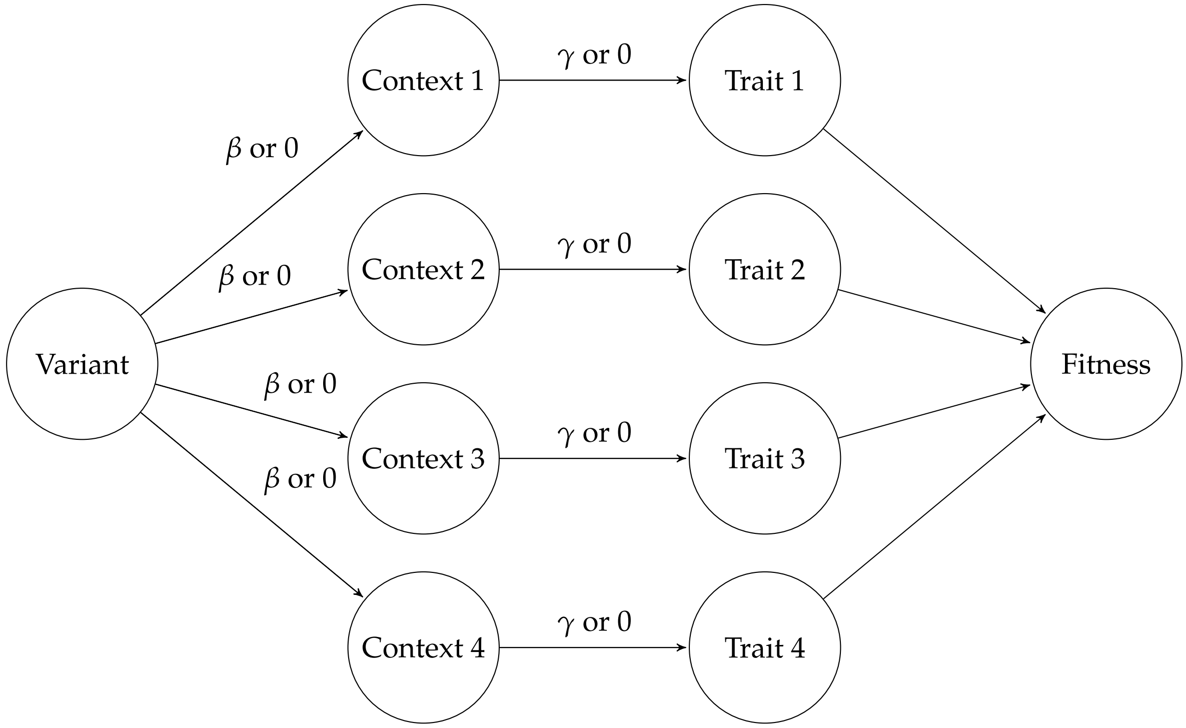

Figure S21: The simplest model we will consider. There is a one-to-one correspondence between contexts and traits. Variants either do or do not affect each context, and for a given gene each context may or may not affect its corresponding trait.

bioRxiv preprint doi: https://doi.org/10.1101/2024.12.12.628073; this version posted December 16, 2024. The copyright holder for this preprint (which was not certified by peer review) is the author/funder, who has granted bioRxiv a license to display the preprint in perpetuity. It is made available under aCC-BY 4.0 International license.

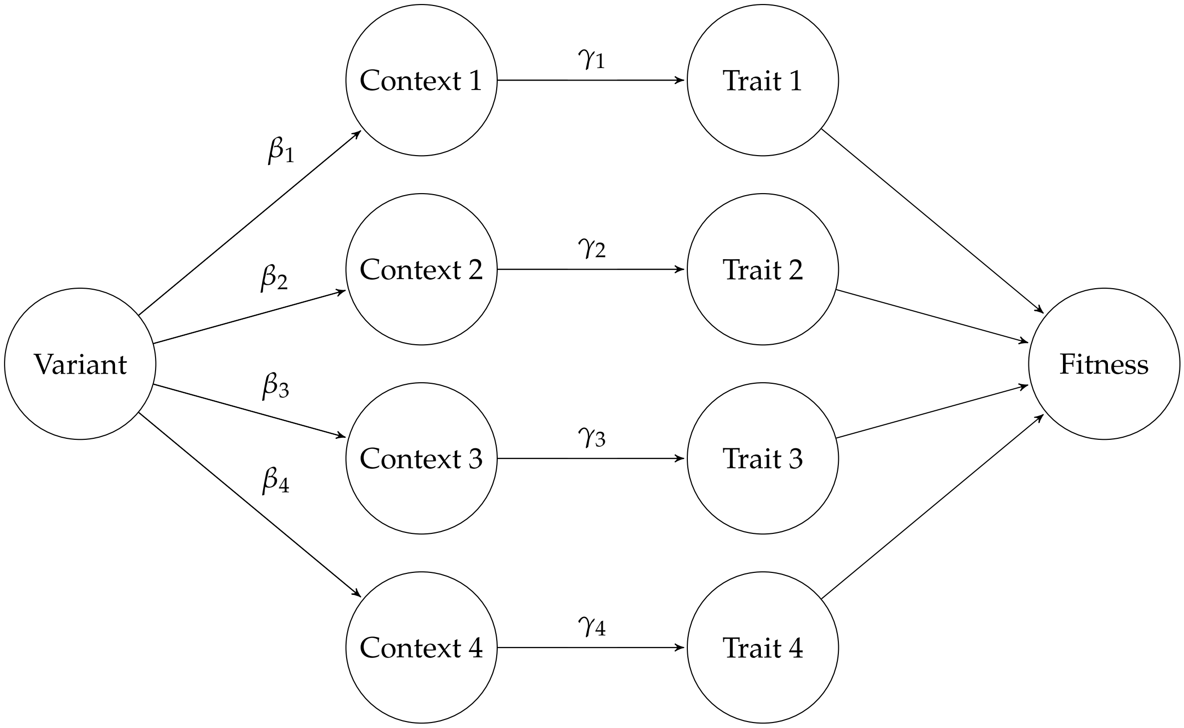

Figure S22: A slight relaxation of the simplest model. There is still a one-to-one correspondence between contexts and traits, but now variants can have arbitrary effects on contexts, and genes can have arbitrary effects on the corresponding trait in each context.
